# Urethane dimethacrylate-based photopolymerizable resins for stereolithography 3D printing: A physicochemical characterisation and biocompatibility evaluation

**DOI:** 10.1007/s13346-023-01391-y

**Published:** 2023-07-15

**Authors:** Giulia Pitzanti, Valentyn Mohylyuk, Francesca Corduas, Niall M. Byrne, Jonathan A. Coulter, Dimitrios A. Lamprou

**Affiliations:** 1 School of Pharmacy, Queen’s University Belfast, Belfast, BT9 7BL UK; 2 Laboratory of Finished Dosage Forms, Faculty of Pharmacy, Riga Stradiņš University, 21 Konsula Street, Riga, 1007 Latvia; 3 Nanotechnology and Integrated Bio-Engineering Centre (NIBEC), Ulster University, Jordanstown Campus, Newtownabbey, BT37 0QB UK

**Keywords:** 3D printing, Photopolymerization, SLA, UV-light curing inks, Dimethacrylate, Biomedical applications

## Abstract

Vat photopolymerisation (VP) three-dimensional printing (3DP) has attracted great attention in many different fields, such as electronics, pharmaceuticals, biomedical devices and tissue engineering. Due to the low availability of biocompatible photocurable resins, its application in the healthcare sector is still limited. In this work, we formulate photocurable resins based on urethane dimethacrylate (UDMA) combined with three different difunctional methacrylic diluents named ethylene glycol dimethacrylate (EGDMA), di(ethylene glycol) dimethacrylate (DEGDMA) or tri(ethylene glycol) dimethacrylate (TEGDMA). The resins were tested for viscosity, thermal behaviour and printability. After printing, the 3D printed specimens were measured with a digital calliper in order to investigate their accuracy to the digital model and tested with FT-IR, TGA and DSC. Their mechanical properties, contact angle, water sorption and biocompatibility were also evaluated. The photopolymerizable formulations investigated in this work achieved promising properties so as to be suitable for tissue engineering and other biomedical applications.

**Graphical abstract:**

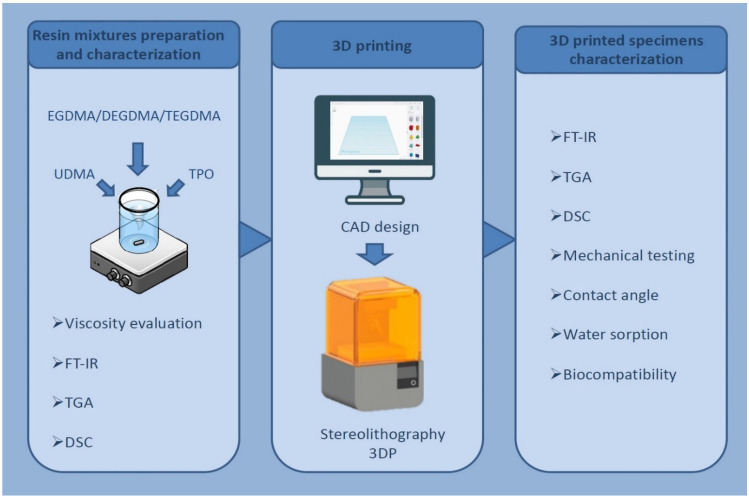

## Introduction

Over the last decade, three-dimensional printing (3DP) has experienced rapid growth and demonstrated great potential in different fields such as bioengineering, pharmaceuticals, microfluidics and electronics [1]. Among the 3DP approaches, the ones based on photopolymerisation such as stereolithography (SLA) and digital light processing (DLP) have attracted attention due to their easiness of use and quick production speed [2]. SLA was the first 3DP technology to be developed and subsequently patented by Charles Hull in 1984. This approach involves layer-by-layer photopolymerisation by crosslinking reactions of a photopolymerizable liquid through the use of a UV laser. Once a layer is polymerised, the building platform is lowered again into the photopolymerizable liquid and the cycle is repeated until the completion of the 3D structure [3, 4]. SLA allows the production of different 3D objects with a high degree of reproducibility offering an accurate final microstructure and geometry. However, it often requires a long time for post-processing and there is a low number of materials appropriate for SLA healthcare applications [3].

The starting material necessary for vat photopolymerisation (VP) 3DP is a mixture of three main components; a monomer or oligomer, which comprises reactive groups essential to build the polymeric network. The most used are acrylate and methacrylate as they are characterised by a fast reactivity. The physical and mechanical properties of the 3DP specimen will be determined by the backbone of the monomer. Another essential component is the photoinitiator, which the main function is to initiate the reaction as a consequence of light absorption. Sometimes a dye or colourant is also added in order to control the light penetration during the 3DP process and ensure high resolution [5].

Numerous photopolymerizable resins have been produced and many are commercially available, with the majority composed of multi-functional monomers based on methacrylate or acrylic esters [6]. The main drawback related to the application of photopolymerisation in the healthcare sector is the lack of availability of FDA-approved biocompatible photopolymerizable materials mainly related to the presence of unreacted products after the polymerisation (monomer, photoinitiator, and additives) [2]. Thus, there is an increasing demand for the development of novel photocurable resins that can be employed in the medical and pharmaceutical sectors [6]. One of the most employed biocompatible materials in VP 3DP is urethane dimethacrylate. Urethane dimethacrylate [7,9,9-trimethyl-4,13-dioxo-3,14-dioxa-5,12-diaza-hexadecane-1,16-diylbis(2-methylacrylate)] is a transparent, odourless, and hydrophobic compound mostly used in the photopolymerisation of dental resins, biomaterials, self-healing materials, and luminescent polymers [7]. For example, in a previous study, diurethane dimethacrylate (UDMA) was mixed with glycerol dimethacrylate (GDMA) and quaternary ammonium methacrylate to obtain an antimicrobial resin suitable for SLA 3DP, with the potential to be used for dental and orthopaedic applications [8]. UDMA possesses a high molecular weight and high viscosity, which could interfere with successful printing. For this reason, it is preferable to combine this monomer with a diluent able to decrease its viscosity and make it more suitable for printing. One of the mostly common diluents employed in association with urethane dimethacrylate is triethylene glycol dimethacrylate (TEGDMA) [9]. The latter is a hydrophilic, low-viscosity, difunctional methacrylic monomer employed as a crosslinking agent [10].

The potential of SLA 3DP in the healthcare sector was investigated by many research groups. SLA 3DP was used to develop different oral dosage forms such as immediate-release tablets, modified-release tablets and tablets containing multiple APIs [11–13]. Moreover, different groups explored its potential in the manufacturing of topical drug delivery systems, such as microneedles and topical films [14–16]. Finally, bio-medical scaffolds, dental prosthetics and medical devices were successfully manufactured by stereolithography 3DP [8, 17–19] .

Most of the previous studies employing SLA 3DP in the development of healthcare products use commercially available photocurable resins. Just few works present an investigation on the formulation. The most studied photocurable polymers in the healthcare sector have been poly (ethylene glycol) diacrylate (PEGDA) [11], poly (ethylene glycol) dimethacrylate (PEGDMA) [20] and poly (propylene fumarate)/diethyl fumarate (PPF/DEF) [21]. Preparing a photopolymerizable formulation by selecting the appropriate raw materials could be convenient leading to a reduction of the costs.

Starting from this background, the aim of this work is to investigate the suitability for SLA 3DP and biocompatibility of novel photopolymerizable resins based on UDMA combined with three different difunctional methacrylic diluents (e.g., tri(ethylene glycol) dimethacrylate—TEGDMA, di(ethylene glycol) dimethacrylate—DEGDMA, and ethylene glycol dimethacrylate—EGDMA). The resins were characterised in terms of viscosity and thermal behaviour. Spectroscopy was employed to evaluate the conversion of the methacrylate groups after printing and after curing. The mechanical properties, water sorption, contact angle and biocompatibility of the 3D-printed specimens were also investigated.

## Materials and methods

### Materials

**Fig. 1:**
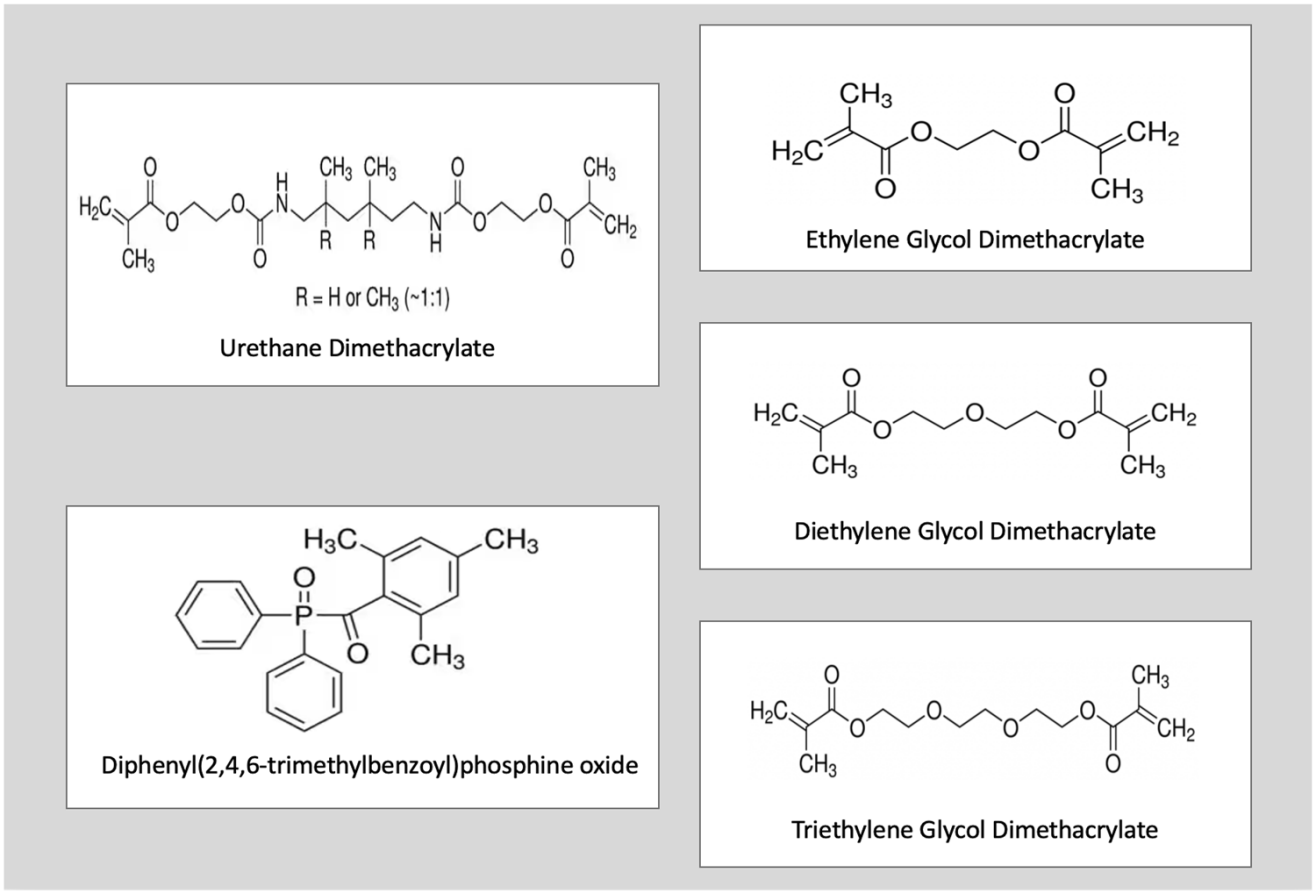
Chemical structures of urethane dimethacrylate, diphenyl(2,4,6-trimethylbenzoyl)phosphine oxide, ethylene glycol dimethacrylate, diethylene glycol dimethacrylate and triethylene glycol dimethacrylate

Urethane dimethacrylate (UDMA), triethylene glycol dimethacrylate (TEGDMA), diethylene glycol dimethacrylate (DEGDMA) and ethylene glycol dimethacrylate (EGDMA) were purchased from Sigma Aldrich (Gillingham, UK) (Fig. 1). Diphenyl(2,4,6-trimethylbenzoyl)phosphine oxide (TPO) was purchased from Tokyo Chemical Industry Co., Ltd. (Tokyo, Japan). Isopropyl alcohol (IPA) was purchased from Sigma-Aldrich (St. Louis, MO, USA). NIH 3T3 embryonic mouse fibroblasts (ATCC-CRL-1658), Dulbecco’s modified Eagle’s medium (DMEM) and calf bovine serum (CBS) were purchased from ATCC (LGC, UK).

### Preparation of the resin mixtures

The resin mixtures were prepared under stirring in dark conditions combining UDMA and one of the co-polymers (EGDMA, DEGDMA, TEGDMA) in equal amounts. In all the mixture, 0.1% w/w of TPO was added as a photoinitiator into the resin mass.

### Viscosity

The viscosity of the resins was assessed in a rotation shear ramp test by using a HAAKE™ MARS™ Rheometer (Thermo Scientific™, UK) with parallel plate geometry. The diameter of the upper and lower plates was 35 mm. The range of shear rate was from 0.1 to 100 s^−1^. The gap between the two geometries was set to 100 µm. The experiments were conducted at room temperature and performed in triplicate for each sample.

### SLA 3D printing

**Fig. 2:**
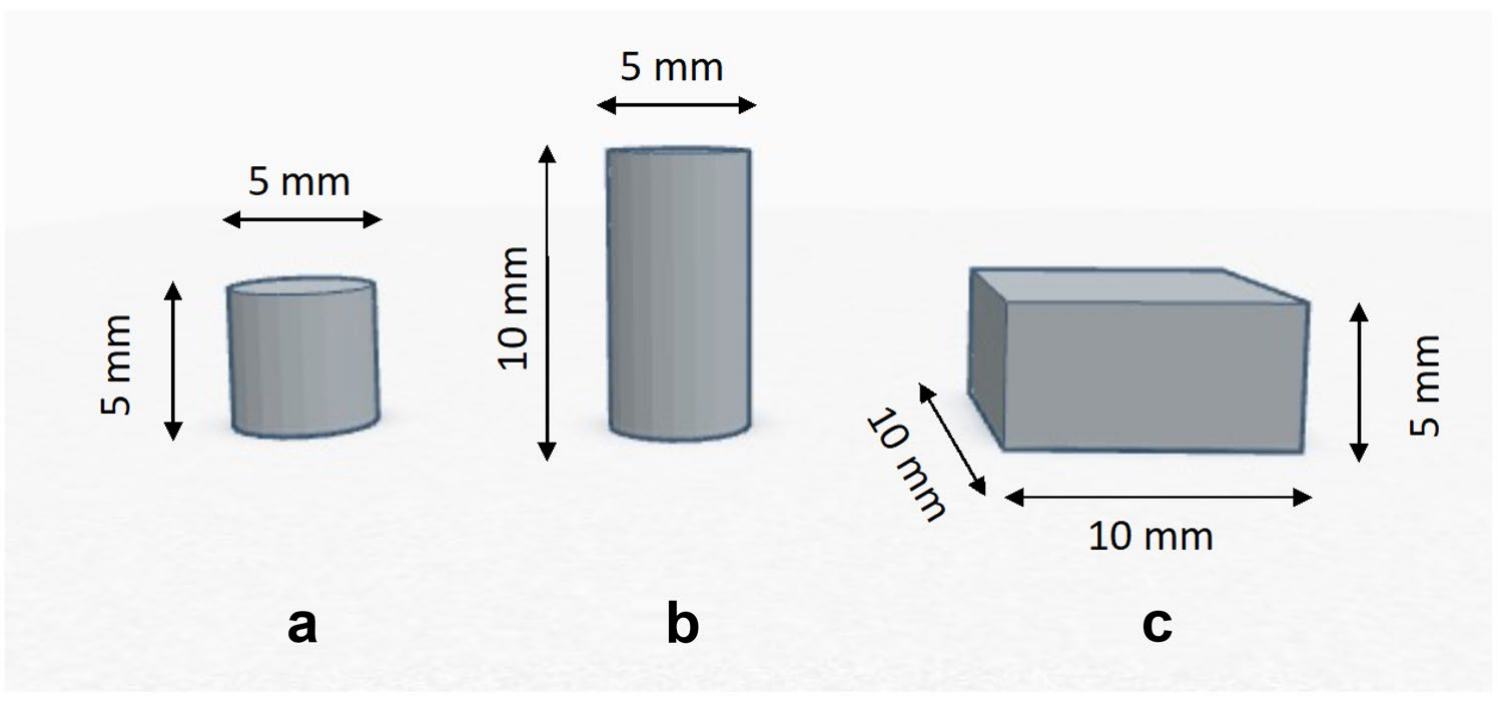
Sketches and CAD sizes of specimens for the printability, biocompatibility (**a**), mechanical properties under compression (**b**), and the contact angle measurement (**c**)

The printability of the resin mixtures was assessed by using the Form 2 desktop 3D printer from Formlabs (Somerville, Massachusetts, USA). Cylindrical samples were produced, aiming to evaluate the accuracy of the digital model, mechanical properties under compression and biocompatibility. Cuboidal samples were printed to measure the contact angle between the printed surface and the wetting agent. The CAD sizes of the samples are reported in Fig. 2.

The designs were prepared by using the computer-aided-design software Tinkercad^®^, exported in stereolithography (.stl) format from Tinkercad to the printer’s Preform software 3.18 and sent to the printer for slicing. The specimens were printed with supports on the build platform. The effect of the printing angle (0°, 15°, 45°, 90°) on the final product was investigated. The open-mode setting was activated to allow disabling the automatic filling of the tank with the photopolymerizable resin. As a material setting, the surgical guide resin was selected. Printing was conducted at room temperature and at a layer thickness of 50 μm. Once the printing process was completed, all the printed prototypes underwent a post-printing treatment. In detail, supports were removed using a side cutter, then the resin in excess was removed by placing the 3DP cylinders into a beaker with 2-propanol, sonicated for 5 min and finally cured under UV (Form Cure, Formlabs; wavelength 405 nm) at room temperature. The effect of different curing times (e.g., 0, 1, 5, 10, 20, and 30 min) was investigated. The accuracies of the printing were evaluated by measuring the height and diameter of the 3D-printed cylinders with a Fisherbrand™ Traceable™ digital calliper (Fisher Scientific, UK) and comparing them with the digital models. All experiments were carried out in triplicate and the results were averaged.

### Fourier-transform infrared spectroscopy (FTIR)

A Nicolet iS50 attenuated total reflection (ATR) FTIR from Thermo Fisher Scientific (Waltham, Massachusetts, USA) was used to investigate any interactions between polymers and the acrylate conversion on both liquid formulations and the 3D-printed parts. The spectra were recorded using ATR mode and collected with a resolution of 4 cm^−1^, averaging 64 scans for each spectrum, in the wavenumbers range of 400–4000 cm^−1^.

### Thermogravimetric analysis (TGA)

The thermal stability of physical mixtures, the 3DP samples not cured, and after 30 min of curing were examined using Thermal Advantage Q50 TGA (TA Instruments, USA). Samples (5–10 mg) were heated in an open aluminium pan at a heating rate of 10 ℃/min from room temperature to 350 ℃. Nitrogen was used as a purge gas at a flow rate of 50 mL/min for all TGA experiments. The weight remaining (%) was plotted as a function of temperature (°C). Based on the first derivative, the values of degradation onset were derived from the original data.

### Differential scanning calorimetry (DSC)

DSC was used to investigate how the different resin mixtures and the 3DP specimens (not cured or cured for 30 min) behave when subjected to thermal stress. The samples (5–10 mg) were weighed into the aluminium pans and crimped with the lid. The analysis was performed using a DSC 214 Polyma (Netzsch, Germany) within a heating range of − 30 to 300 ℃, at a heating rate of 10 ℃/min under nitrogen purge of 40 mL/min. The data were collected with the Proteus thermal analysis software ver. 8.0 (Netzsch, Germany).

### Mechanical testing

**Fig. 3:**
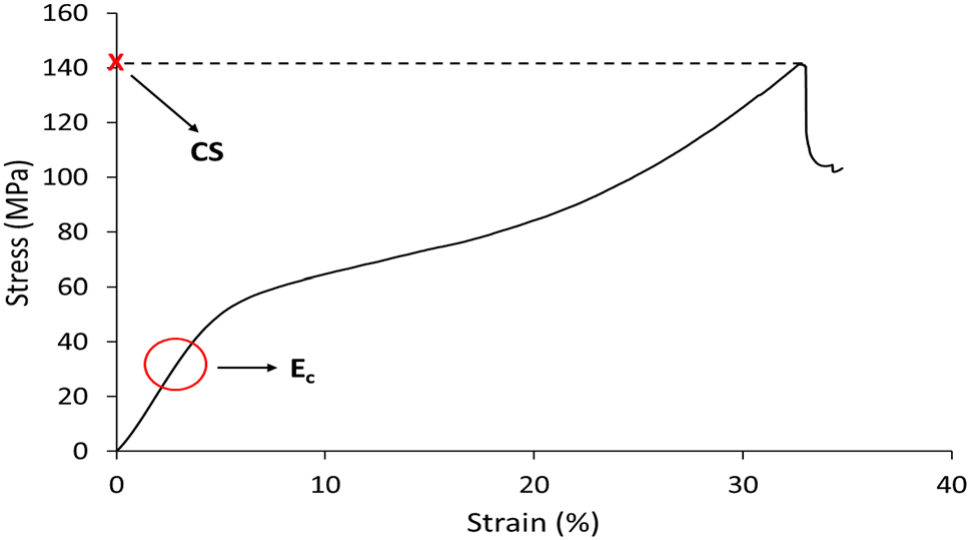
Typical stress–strain curve in compression test. E_c_ and CS are highlighted with a circle and a cross, respectively

Uniaxial compressive tests were performed aiming to evaluate the mechanical response of the 3D-printed specimens. UDMA:EGDMA, UDMA:DEGDMA, and UDMA:TEGDMA samples (*d* = 5 mm, *h* = 10 mm, *n* = 5) were tested using a universal testing machine (Instron 5500S, Instron, UK) equipped with a 5-kN load cell. Tests were carried out using a crosshead speed of 0.5 mm/min until failure. From the raw data, the compressive modulus (EC) and compressive strength (CS) were evaluated, the first as the slope of the stress–strain curve, and the second as the maximum registered stress value before failure (Fig. 3).

### Contact angle

A Biolin Theta Tensiometer (Manchester, UK) was used to determine the surface contact angle. The experiments were conducted at room temperature. Before each experiment, the horizontality of the surface was checked. Five microliters of deionised water was dropped on the surface of the 3DP specimens. The contact angle was registered for 60 s after the drop placement. Contact angle measurements were taken as the mean contact angle registered during 60 s by using the One-Attension software (version 1.8 Biolin Scientific). Five specimens of each resin mixture were tested.

### Water sorption

Water sorption was measured according to ISO 4049 [22]. Before testing, cylindrical specimens (5 × 5 mm) were dried in an oven at 37 ℃ until the constant weight and measured with calliper to determine the initial volume (V). The initial weight (m_0_) was recorded with analytical balance with 0.01 mg accuracy (Fisher Scientific, Loughborough, UK). The specimens were then immersed in distilled water and placed in an oven at 37 ℃ for 1 week. Then, the samples were put out, blotted dry and weighed (m_1_). Water sorption (WS) was calculated using Eq. 1.

1 $$WS (\frac{\mu g}{{mm}^{3}})= \frac{{m}_{1}-{m}_{0}}{V}$$

### Biocompatibility

NIH 3T3 was cultured in DMEM containing 10% v/v CBS at 37 °C in 5% CO_2_-humidified air. Cells were routinely tested for *mycoplasma* and were verified to be negative using the MycoAlert Mycoplasma Detection Kit (Lonza, UK). The cytotoxicity of the printed resin specimens was evaluated in accordance with the ISO 10993–5. Extracts of the printed cylinders (5 × 5 mm; 0.15 g ± 0.01 g) were obtained by incubating in a 2-ml complete medium (DMEM + 10% CBS) at 37 ℃ for 24 h under continuous stirring (90 rpm). Concurrently, NIH 3T3 cells were seeded into each well of a 96-well plate (10,000 cells/well) and incubated for 24 h. Following incubation, the cell culture medium was replaced with 200 µl of controls (complete media-only or 10% DMSO) or 200 µl of sample extracts at 100% or 50% concentrations and incubated at 37 °C for 24 h. Following exposure to extracts, cell viability was analysed by alamarBlue in accordance with manufactures instructions (Thermo Scientific, UK). Briefly, a 10% alamarBlue solution was added to each well and plates incubated at 37 °C for 4 h before fluorescence was read (Ex. 544; Em. 590) using a FLUOstar Omega microplate reader (BMG Labtech, Germany). Initially, a control well (no cells) containing media + alamarBlue was used to measure and subtract background fluorescence from experimental wells (containing cells). Subsequently, results were normalised relative to that of the negative controls (complete media-only). Experiments were performed in triplicate.

### Statistical analysis

Data are presented as mean ± standard deviation. Experiments were conducted using a minimum of three replicates (*n* ≥ 3). The statistical analysis was performed via one-way analysis of variance (ANOVA), and *p* < 0.05 was considered statistically significant.

## Results and discussion

**Fig. 4:**
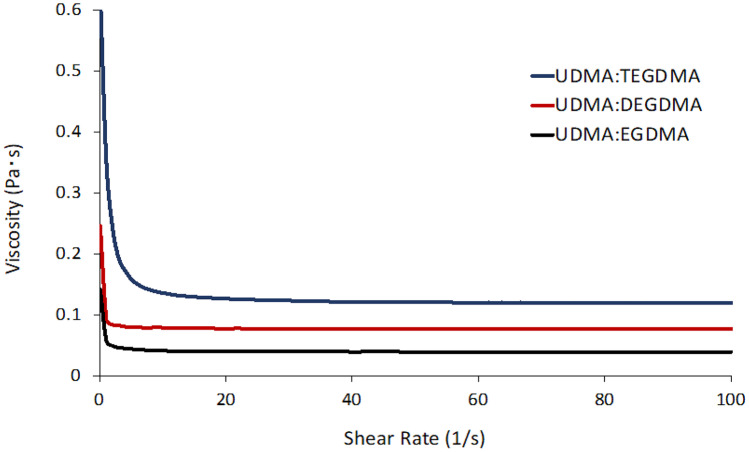
Viscosities of formulations under continuous shear rate sweep

One key factor for a photopolymerizable resin to be printable is its viscosity. It has been reported that the suitable viscosity values of a resin for SLA or DLP printing are up to 10^3^ mPa·s [23]. Highly viscous resins could be obstacles to the manufacturing process of the objects during layer-to-layer printing. Indeed, viscosity plays a role in adhesion to the build plate in bottom-up photo-curing 3DP systems. Therefore, photopolymerizable resins characterised by a relatively low viscosity allow the manufacturing of 3D objects in a fast and accurate manner [5]. In order to assess the suitability of the resins for SLA 3DP, a preliminary study investigating viscosity was performed. Figure 4 shows the rheological behaviour of the three photopolymerizable resins. It can be observed that the viscosity of the mixture was dependent on the EGDMA derivative used in combination with UDMA. The viscosity increased along with the ethylene glycol groups increase. In particular, at low shear rates, the viscosity quickly decreased until reaching a plateau at around 15 s^−1^ for the resin UDMA:TEGDMA and at around 5 s^−1^ for the mixtures UDMA:EGDMA and UDMA:DEGDMA. Overall, when UDMA was combined with TEGDMA, the highest viscosity was observed, whereas when associated with EGDMA, the lowest viscosity of the three tested mixtures was obtained. However, all the three mixtures showed suitable viscosity values for SLA printing.

**Fig. 5:**
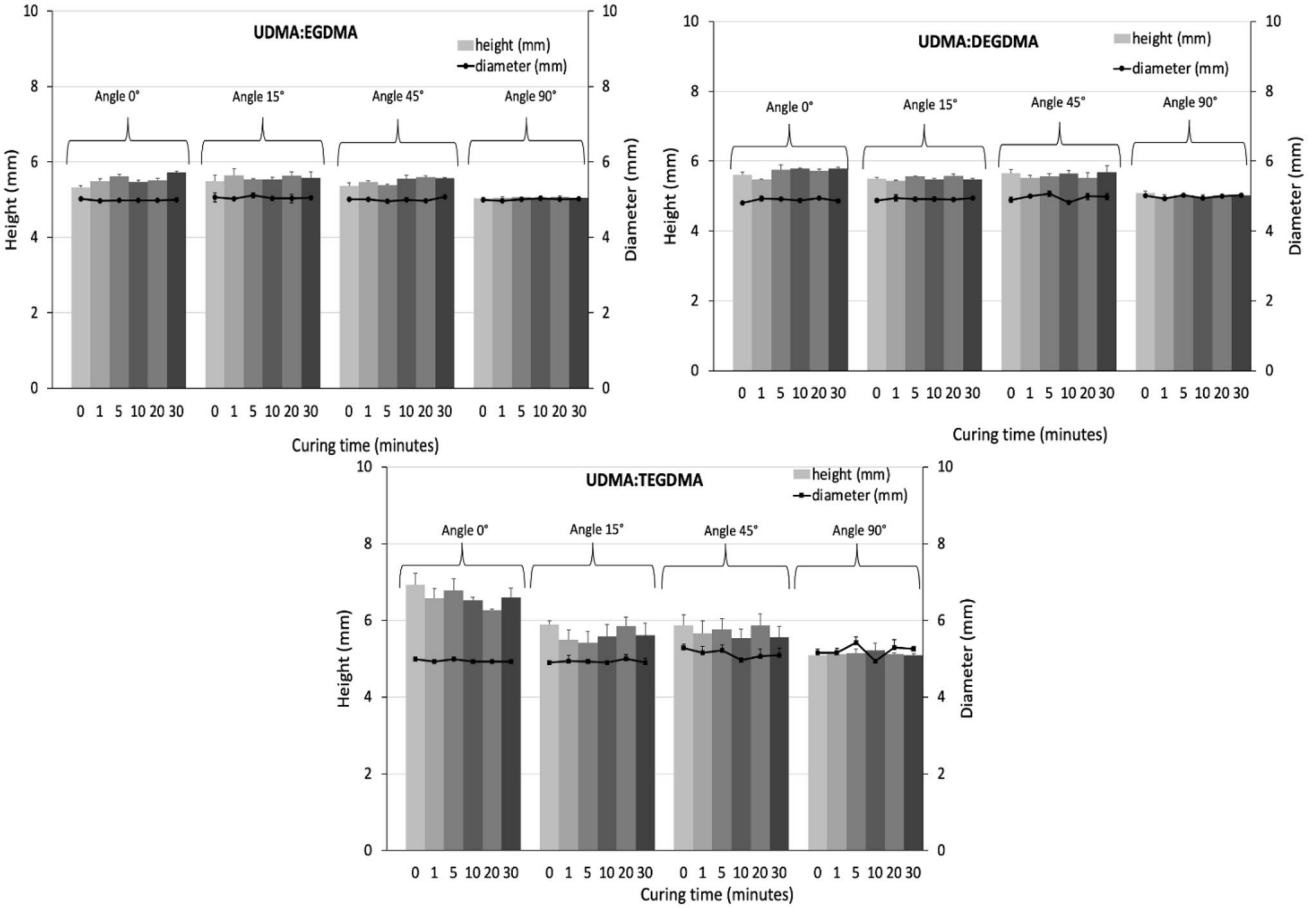
Effect of printing angle and curing time on height and diameter of cylindrical specimens (5 × 5 mm)

Once the suitability of the resins’ viscosity for SLA 3DP was assessed, they were employed for the 3DP of cylindrical specimens with 5-mm diameter and 5-mm height. An investigation on the effect of the printing angle and curing time on the accuracy of the final object to the CAD model was performed (Fig. 5).

For all the resin mixtures, it was observed that by increasing the printing angle, a relatively higher fidelity to the digital model could be obtained. The height of the specimen was statistically more affected (*p* < 0.05) by the changing of the printing angle than the diameter that remained generally constant (*p* > 0.05). Curing time seemed to not affect the accuracy (*p* > 0.05). Among all the formulations, the 3DP cylinders fabricated starting with the resin mixture composed of UDMA and EGDMA showed the highest and approximately the same accuracy as the initial design. While the lowest accuracy of TEGDMA samples can be explained by their relatively higher viscosity compared to the other resin mixtures formulated in this work.

**Fig. 6:**
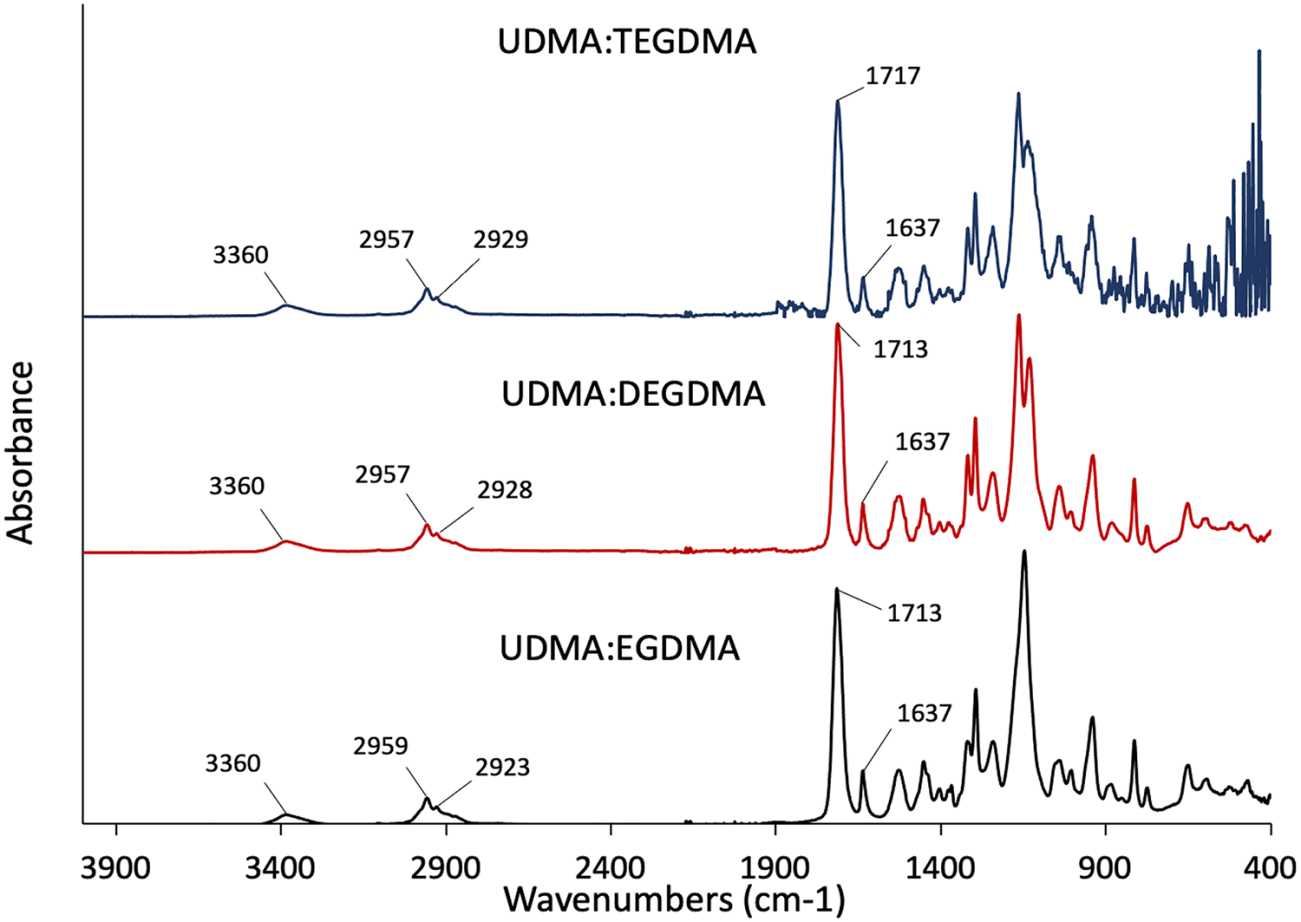
FT-IR spectra of the three resin mixtures before printing

FT-IR was used to investigate the spectra of the resin mixture and to observe the changes during the photopolymerisation process (Fig. 6).

The FTIR spectra of the resins showed a peek at 3360 cm^−1^ corresponding to the NH group of urethanes. Between 2923 and 2959 cm^−1^, the peaks related to the C–H stretching can be observed. At 1713 cm^−1^ the peak of the C = O has been observed. Peaks at 1637 cm^−1^ are attributed to the methacrylic group [24].

**Fig. 7:**
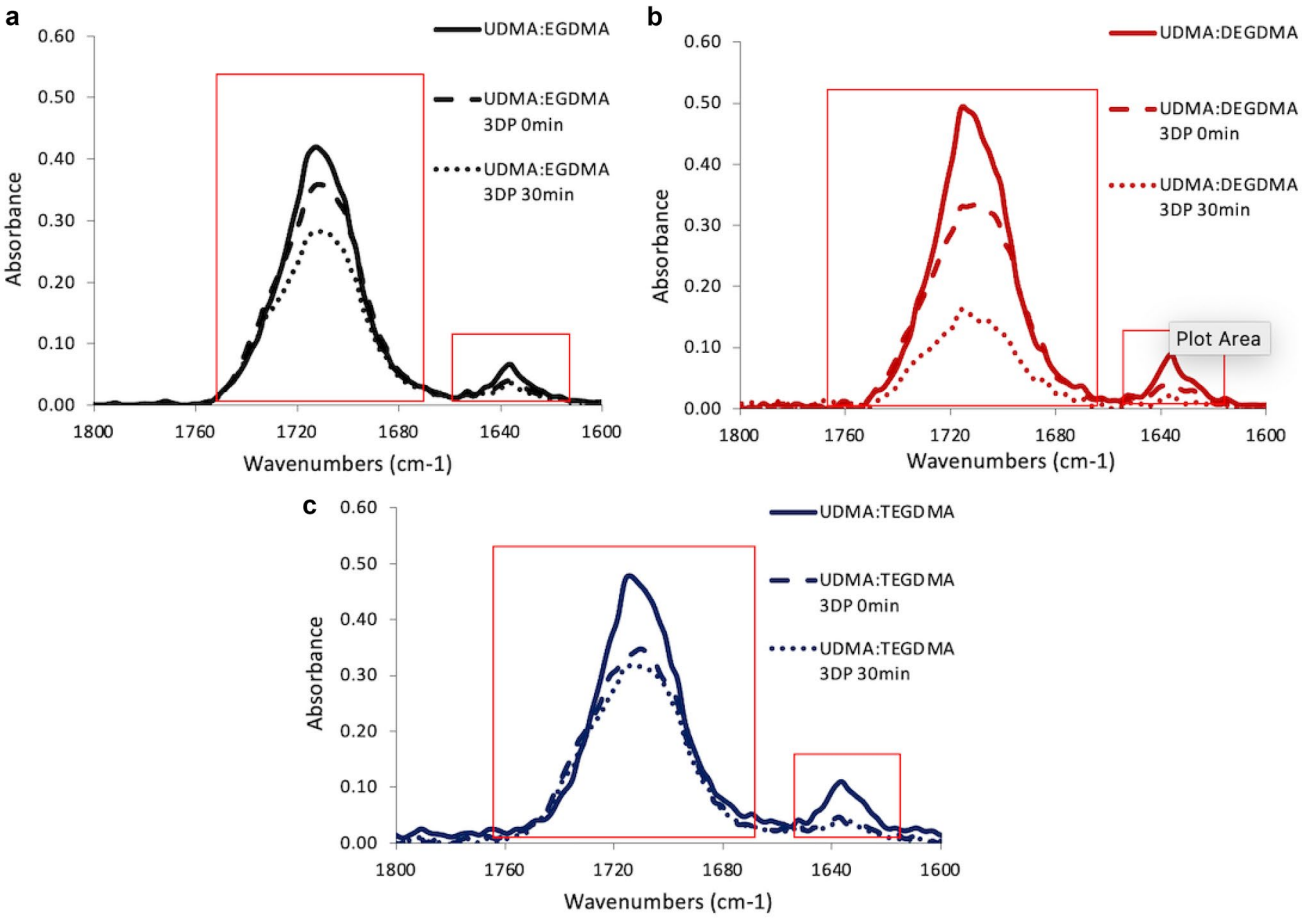
FT-IR spectra (between 1600 and 1800 cm^−1^) of the resins and of the 3DP specimens after printing and after 30 min of UV curing: UDMA: EGDMA (**a**), UDMA:DEGDMA (**b**) and UDMA:TEGDMA (**c**)

To observe the changes of FTIR profiles during the photopolymerisation process, the spectra obtained from each resin mixture at three time points were compared: before printing, after SLA 3DP and after the maximum curing time employed in this work (30 min). The extent of chemical cross-linking is an important parameter since it affects the physicochemical and mechanical properties of the system [25]. Moreover, it depends on the number of free monomers which could have an influence on the biocompatibility of the system; Fig. 7 shows the results of this study. For all three resins, after SLA 3DP, a decrease in the intensity of the band corresponding to the C = C scissoring at 1637 cm^−1^ was observed. This is due to the fact that C = C bonds in methacrylate groups are involved in the photopolymerisation reaction. Moreover, a decrease of the peak at 1713 cm^−1^ related to the C = O of the ester groups was also observed. The latter is a consequence of electronic conjugation [3].

**Fig. 8:**
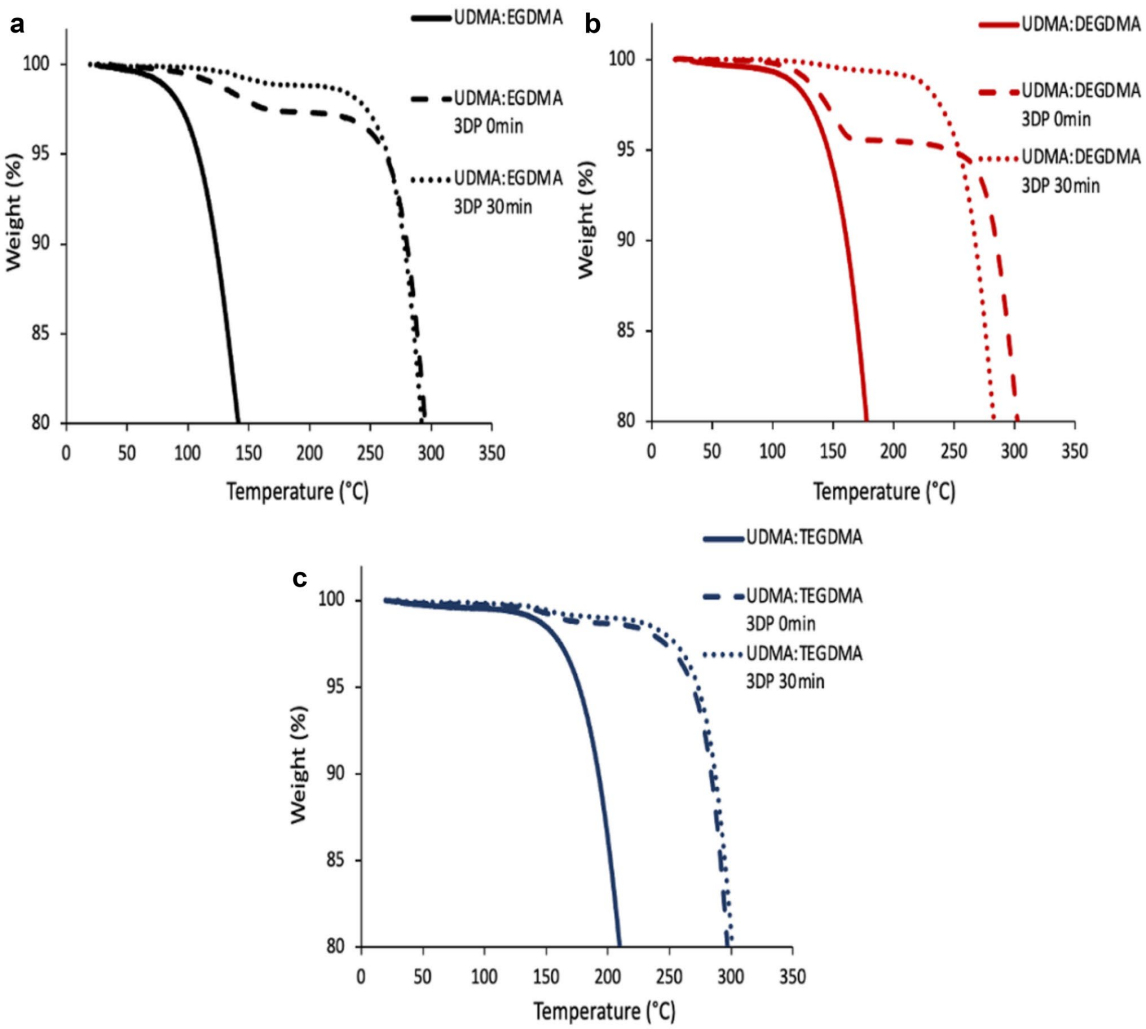
TGA of the resins and of the 3DP specimens after printing and after 30 min of UV curing: UDMA: EGDMA (**a**), UDMA:DEGDMA (**b**) and UDMA:TEGDMA (**c**)

A different number of thermal events for each physical mixture of liquid resins compared to the 3DP and 3DP and cured samples was determined by TGA (Fig. 8). Indeed, an evaporation event only was observed for the liquid resins, whereas in 3DP samples, two thermal events were observed. The first thermal event was attributed to the evaporation of volatile unpolymerized resins [26]. The physical mixtures of resins were fast evaporated along with the temperature increase. For the physical mixtures of non-crosslinked resins, an increase in the evaporation onset temperature was observed in the following order UDMA:EGDMA, UDMA:DEGDMA and UDMA:TEGDMA. The evaporation from all printed samples was a step-like event that started at a temperature of more than 100 ℃ and comprised not more than 2% of weight loss except for 4.8 ± 0.6% of weight loss for UDMA:DEGDMA uncured 3DP sample. Crosslinked resins—3DP samples as well as 3DP and cured samples compared to the liquid resin showed limited evaporation (which can be attributed to non-crosslinked fraction) followed by degradation event. Indeed, the degradation onset of all 3D-printed samples was relatively the same and higher than 260 °C. Compared to the values obtained straight after printing, curing almost did not change the onset of degradation. The TGA profiles of the physical mixture of resins, 3DP and of 3DP and cured samples were in agreement with the literature [26, 27].

The printing process was carried out at room temperature; therefore, the degradation temperature of the materials determined by TGA was not overcome.

**Fig. 9:**
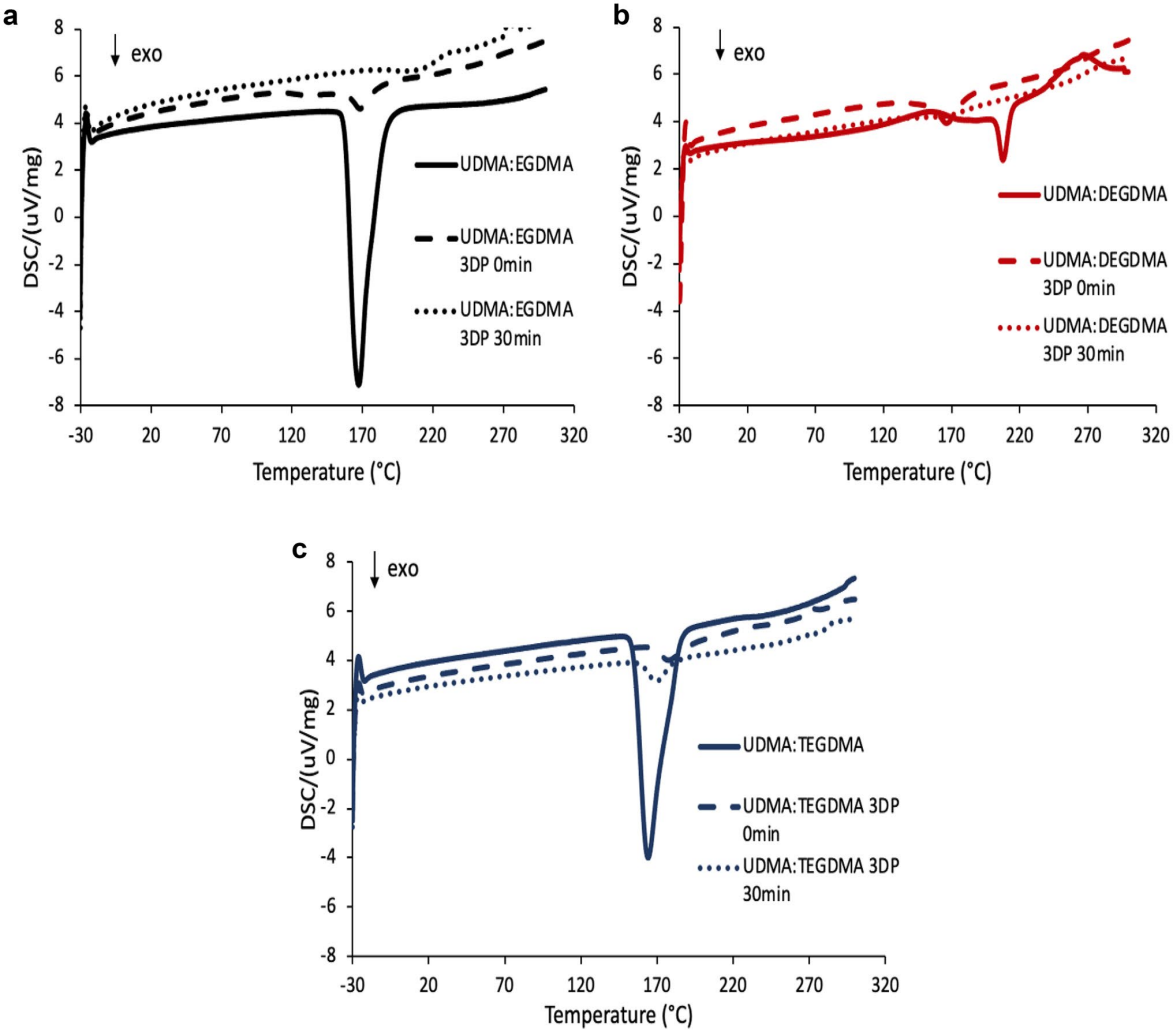
DSC of the resins and of the 3DP specimens after printing and after 30 min of UV curing: UDMA: EGDMA (**a**), UDMA:DEGDMA (**b**) and UDMA:TEGDMA (**c**)

A further thermal investigation on the different resin mixtures and on the 3DP samples not cured and after 30 min of curing was performed by DSC. The results of the experiments are reported in Fig. 8. As shown in previous studies on urethane dimethacrylate polymer, the DSC-observed exothermic peak was attributed to a reaction of polymerisation [28–30]. All sets of samples demonstrated a decrease in the exothermic peak area from the physical mixture of resins to printed uncured and then to the printed and 30-min cured sample. We need to point out that during the SLA printing process, the components of the resin mixtures are involved in a radical photopolymerization; therefore, it is not surprising that after printing and after curing, the residual polymerizable components are lower. A decrease in the intensity of DSC exothermic peaks for cured samples compared to the liquid resins was also observed in previous studies [31]. The thermal events on the DSC curves after 260 °C were related to the degradation process because they are situated after the degradation temperature onset determined with TGA (Fig. 9).

The influence of the employed materials mixtures and curing times on the specimens’ mechanical properties was investigated via uniaxial compression test.

Among the tested mixtures and for a curing time of 0 min, UDMA:EGDMA was characterised by the highest E_C_, followed by UDMA:TEGDMA and UDMA:DEGDMA (*p* < 0.05). Although UDMA:EGDMA proved to be stiffer than the other material mixtures, UDMA:TEGDMA was characterised by the highest CS, therefore being able to withstand higher compressive loads when compared to the other produced mixture (*p* < 0.005).

**Fig. 10:**
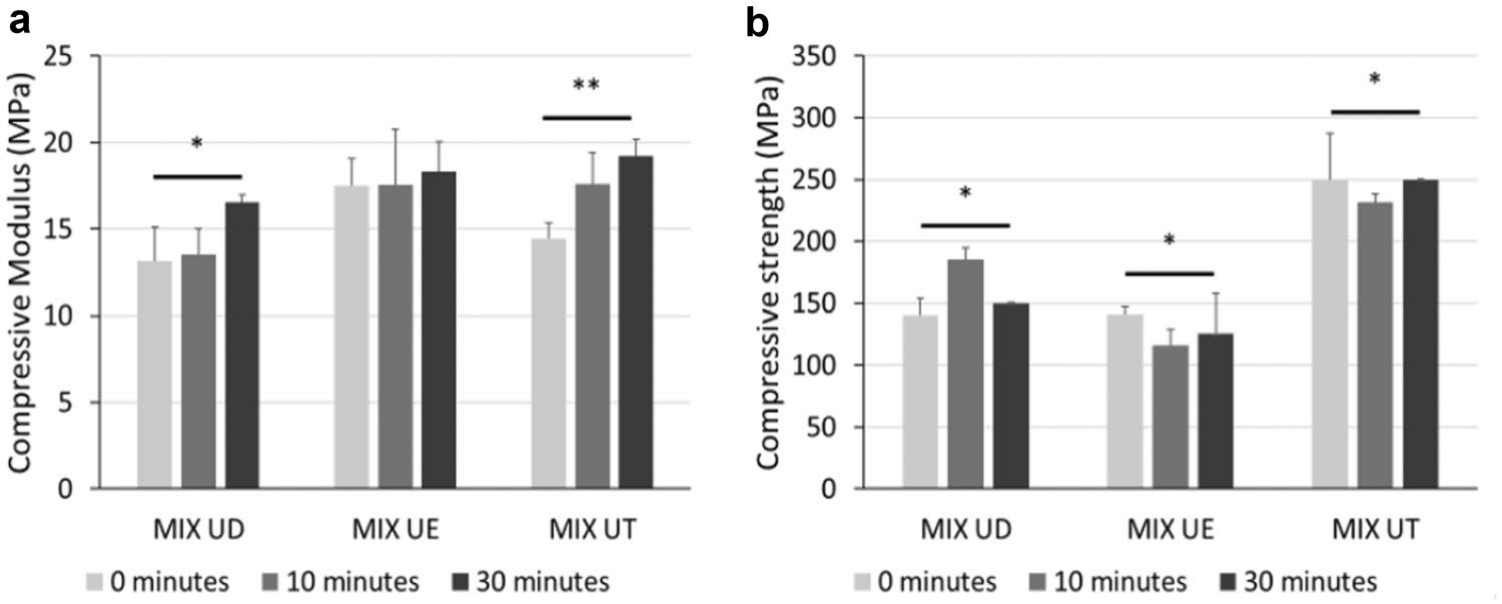
Mechanical properties trend for each tested mixtures and before and after the curing process. **a** Compressive modulus and **b** compressive strength (*p* < 0.5*; *p* < 0.05**)

As reported in Fig. 10a, longer curing times caused a general increase of the E_C_ values in all the samples, with the highest increase observed for UDMA:TEGDMA and followed by UDMA:DEGDMA after 30 min of curing time, meaning that samples were stiffer after curing. This behaviour was generally in accordance with what has been previously reported in a work by Riccio et al., where the change in mechanical properties, under tensile conditions, for different commercial-graded resins printed via SLA was evaluated prior and after the curing process. Here, a general stiffening of the materials tested was observed as a consequence of the curing process [32].

Conversely, no clear trend was observed regarding CS (Fig. 10b), as UDMA:TEGDMA’s CS did not vary significantly after the curing process, whereas CS values increased for UDMA:DEGDMA and decreased for UDMA:EGDMA after 30 min of curing.

Overall, it can be inferred that for the material mixtures tested in this research work, the curing time had a significant impact on the final mechanical outcome.

**Fig. 11:**
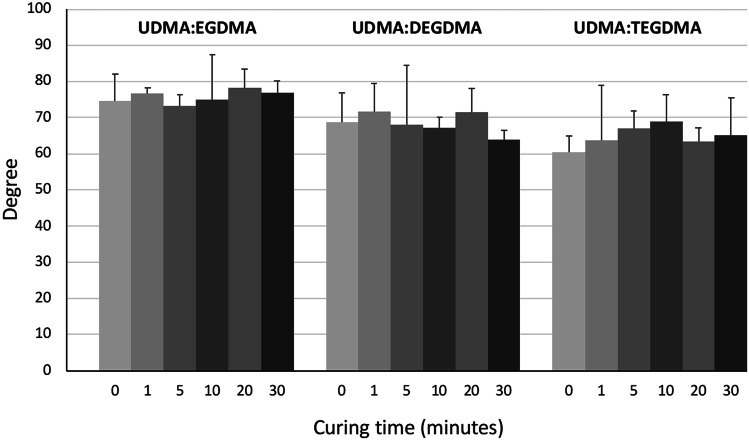
Contact angle of the 3DP cubes made from the three different resin mixtures, exposed to different curing times

Figure 11 shows the contact angle measurements of the 3DP rectangular prisms made from the three resin mixtures and exposes to different curing times. Overall, contact angles were comprised of between 60 and 78°. UDMA:TEGDMA showed the lowest degree among the three resin mixtures, while the highest values were obtained for UDMA:EGDMA. An increase of the curing time did not determine a statistically significant variation of the contact angle. Indeed, a *p* value > 0.05 was obtained for all the resin mixtures. Depending on the water contact angle measurements, the materials can be defined as hydrophilic or hydrophobic. It is well-known that when the contact angle is higher than 90°, the surface is recognised as hydrophobic, while when the contact angle is lower than 90°, the surface is considered as hydrophilic [33, 34]. Contact angle measurement provides a first determination on the suitability of the material for a specific biomedical application. In particular, it is recognised that hydrophilic surfaces are able to limit cell adhesion or blood platelet activation which should enhance biocompatibility. These types of surfaces are desirable for short-term implants but are not suitable for applications where cell adhesion is desired [33]. A proper equilibrium between hydrophilicity and hydrophobicity is required for blood-contacting devices and tissue-engineering substrates. Indeed, highly hydrophobic surfaces enhance cell adhesion and reduce biocompatibility, but highly hydrophilic surfaces avoid cell–cell interactions [35].

The ability to absorb water is an important parameter to consider for the system that will be in contact with the physiologic media [36]. Water molecules can infiltrate and diffuse within the polymeric macrostructure leading to a degradation and break of the chemical bonds which influence the polymer solubility. Moreover, it can make the material softer, reduce its strength and flexibility and cause deformation [37]. Therefore, it is desirable that water sorption is as low as possible.

**Fig. 12:**
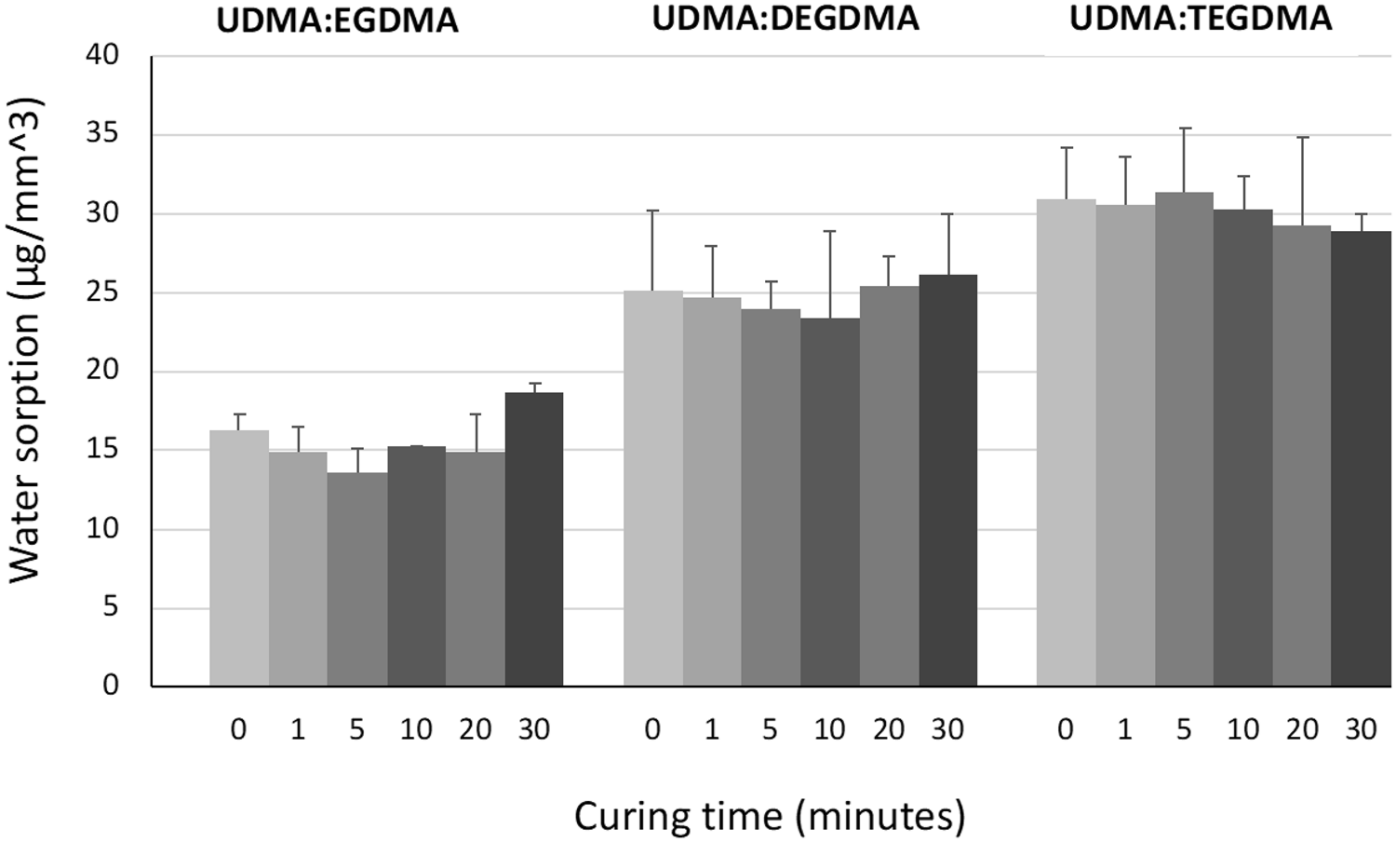
Water sorption of the 3DP specimens made from the three different resin mixtures, exposed to different curing times

In our work, water sorption ranged between 14 and 31 μg/mm^3^ (Fig. 12). In detail, specimens 3DP from the resin UDMA:EGDMA showed water sorption values between 14 and 19 μg/mm^3^ whereas specimens 3DP from the resin UDMA:DEGDMA showed values between 23 and 26 μg/mm^3^. The highest water sorption values were obtained with the ones produced from the resin UDMA:TEGDMA (between 29 and 31 μg/mm^3^). All the values were lower than those required by the ISO 4049 standard, which establishes that the maximum water sorption value is 40 μg/mm^3^. Overall, the type of the resin mixture had a significant influence on the water sorption (*p* < 0.05) whereas an increase of the curing time did not determine a statistically significant variation of the water sorption ability (*p* > 0.05).

**Fig. 13:**
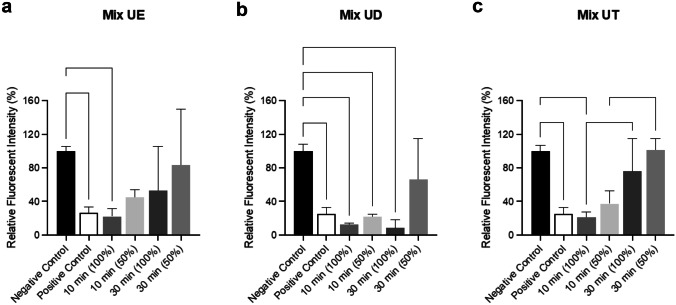
Viability of NIH 3T3 cells following treatment (24 h) with 100% and 50% extracts from **a** Mix UE, **b** Mix UD and **c** Mix UT at 10-min and 30-min UV curing times in comparison to negative (media-only) and positive (10% DMSO) controls, as analysed by alamarBlue. Values are shown for *n* = 3 (Bars indicate mean with SD. Statistical significance determined by one-way ANOVA with Tukey’s multiple comparison test)

The biocompatibility of resin mixtures in vitro was determined using alamarBlue (Fig. 13), given its increased sensitivity in comparison to the MTT and XTT assays [38, 39]. Treatment with 100% extracts from resins UV cured for 10 min had a significant impact on NIH 3T3 cell viability in comparison to control reducing viability to 22.2% ± 9.1% (Mix UE, *p* = 0.0139; Fig. 13a), 12.9% ± 1.3% (Mix UD, *p* < 0.0001; Fig. 13b) and 20.3% ± 6.3% (Mix UT, *p* < 0.0001; Fig. 13c). Similarly, 100% extracts from Mix UD resins cured for 30 min significantly reduced cell viability to 8.5% ± 9.7% (*p* < 0.0001; Fig. 13B) while 100% extracts from Mix UE resins reduced cell viability to 53.1% ± 52.5 (Fig. 13A). Interestingly, for Mix UT resins, longer UV curing times (30 min) significantly improved biocompatibility in vitro in comparison to 10-min curing (*p* = 0.0052), reducing viability to 75.9% ± 39.3% (Fig. 13c). Furthermore, treatment with the 50% extract from Mix UT resins had no impact on NIH 3T3 cell viability (101.5% ± 13.3%). Given that the reduction in viability of NIH 3T3 cells treated with the highest concentration of the 30-min cured Mix UT resin extract is less than 30%, the material can be considered as non-cytotoxic in line with recommendations from ISO 10993–5.

## Conclusion

This study investigated the suitability of photocurable resins based on urethane dimethacrylate combined with three different difunctional methacrylic diluents named tri(ethylene glycol) dimethacrylate, di(ethylene glycol) dimethacrylate or ethylene glycol dimethacrylate for stereolithography 3DP. All the resins demonstrated a suitable viscosity for SLA printing. The effect of the printing angle on the accuracy of the computer-aided-design was evaluated. It was figured out that by increasing the printing angle, a higher fidelity to the digital model could be obtained. Curing time seemed to not affect the accuracy of the 3DP specimens to the CAD design. 3DP cylinders made by the resin mixture composed of UDMA and EGDMA showed the highest accuracy to the initial design. The mechanical studies demonstrated that 3DP specimens based on the mixture UDMA:TEGDMA were the most resistant to compressive failure with respect of all the other mixtures and possessed the lowest contact angle and the highest water sorption.

Overall, the newly developed resins for SLA 3DP showed promising properties so as to be suitable for tissue engineering and other biomedical application. In future works, it would be interesting to mix them with drugs and tests for various types of drug delivery systems such as tablets, capsules, microneedles, drug-eluting implants, and scaffolds. Moreover, the study has provided a low-cost alternative to commercially available SLA photocurable resins used in previous studies of SLA 3DP in drug delivery.

## Data Availability

The datasets generated during and/or analysed during the current study are available from the corresponding author on reasonable request.
